# The complete mitochondrial genome of *Fusicolla acetilerea* (Nectriaceae, Hypocreales)

**DOI:** 10.1080/23802359.2026.2630474

**Published:** 2026-02-16

**Authors:** Yehyeon Cha, Young-Hyun You, Seung-Yeol Lee, Hee-Young Jung, Changmu Kim, Seung-Yoon Oh

**Affiliations:** ^a^ Department of Biology and Microbiology, Changwon National University, Changwon, Republic of Korea; ^b^ Species Diversity Research Division, National Institute of Biological Resources, Incheon, Republic of Korea; ^c^ Department of Plant Medicine, Kyungpook National University, Daegu, Republic of Korea; ^d^ Institute of Plant Medicine, Kyungpook National University, Daegu, Republic of Korea; ^e^ School of Advanced Biosciences, Changwon National University, Changwon, Republic of Korea

**Keywords:** Fungi, mitogenome, Nectriaceae, phylogenetic analysis

## Abstract

*Fusicolla acetilerea* (Nectriaceae, Ascomycota) is an emerging saprotrophic/plant-associated fungus whose genomic resources remain scarce. Here, we report the complete mitochondrial genome of *F. acetilerea* strain NIBRFGC000505922, assembled from Illumina NovaSeq reads. The circular-mapping mitogenome is 29,182 bp in length with a GC content of 28.7%. It encodes the canonical set of oxidative phosphorylation genes, two rRNA genes, 25 tRNA genes, and an intronic *rps3* gene. A phylogenetic analysis based on 14 core protein-coding genes places *F. acetilerea* within Nectriaceae, forming a well-supported clade with *Nectria cinnabarina*. This mitogenome provides a reference for various genetic studies in *Fusicolla* and related taxa.

## Introduction

The genus *Fusicolla* Bonord. 1851 is primarily saprotrophic and has been detected on diverse organic substrates, including decaying twigs (Zeng and Zhuang 2023), soil (Liu et al. 2022), wild-boar bones (Lechat and Rossman 2017), and even water (Jeon et al. 2020). Beyond its ecological roles, multiple studies have emphasized the significance of *Fusicolla* species on human health (Zhong et al. 2021), fermentation (Zhu and Huang 2021), and agriculture (Wang et al. 2020). Given these contexts, research on the diversity and functional traits of *Fusicolla* is of both academical and practical importance.

*Fusicolla acetilerea* (Tubaki, C. Booth & T. Harada) Gräfenhan & Seifert 2011 (Gräfenhan et al. 2011) is notable species within the genus, characterized by large macroconidia with typically three (occasionally four) transverse septa, formed on phialidic conidiophores, with conidial dimensions of (30–)35–40 × 3.5(–4.0) µm (Tubaki et al. 1976). *F. acetilerea* has been reported from soil, decayed wood, and in association with ambrosia beetles (Biedermann et al. 2013; Ding et al. 2015). Notably, *F. acetilerea* strain NIBRFGC000505922 (=KNUF-20-PBU01), a isolate from soil in South Korea, shows the ability of polycaprolactone (PCL) and polylactic acid (PLA) degradation, which suggests potential application on plastic-waste treatment (Lee et al. 2021). In addition, this species can utilize triacrylonitrile and 4-N-trimethylamino-1-butanol as the sole carbon and nitrogen source (Asano et al. 1981; Fujimitsu et al. 2016).

While nuclear genes (*acl1*, ITS, LSU, *rpb2*, and *tub2*) have been actively used to ecological and phylogenetic studies (Lee et al. 2021; Zeng and Zhuang 2023), none of studies use mitochondrial genome for *Fusicolla* species. In this study, we assembled the mitochondrial genome of *F. acetilerea* strain NIBRFGC000505922 for the first time in this genus and analyzed its phylogenetic relationships with other Nectriaceae species.

## Materials and methods

**Figure 1.**
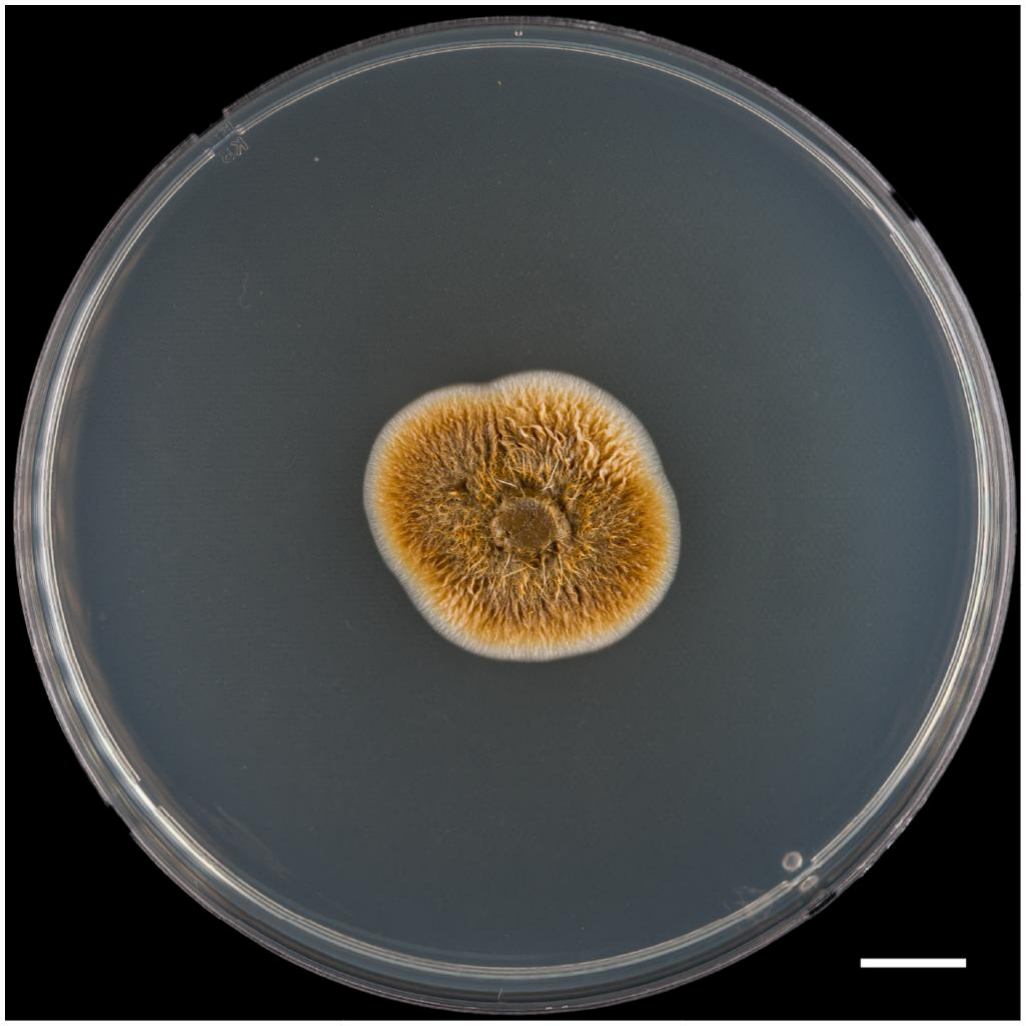
Colony morphology of *Fusicolla acetilerea* strain NIBRFGC000505922 on potato dextrose agar. Scale bar = 1 cm. Photograph by Dr. Jung-Jae Woo.

The *F. acetilerea* strain NIBRFGC000505922 was isolated from the rhizosphere soil associated with pine tree in Boeun, South Korea (36.488767 N, 127.7174 E) and cultured on potato dextrose agar (PDA) at 25 °C (Figure 1). The strain was identified in the previous study (Lee et al. 2021) and is deposited at the National Institute of Biological Resources (NIBR), Incheon, South Korea (https://species.nibr.go.kr/nibrbiobank, contact: nibrbiobank@korea.kr) under the strain number NIBRFGC000505922. Genomic DNA was extracted using the Wizard^®^ HMW DNA Extraction Kit (Promega, Madison, WI). DNA quality was evaluated using a Qubit 4.0 Fluorometer (Thermo Fisher Scientific, Waltham, MA) and TapeStation D1000 ScreenTape system (Agilent, Santa Clara, CA). Sequencing was performed using the Illumina NovaSeqX platform at PHYZEN (Seongnam, South Korea).

Raw sequencing reads were quality-filtered using FASTP (Chen et al. 2018) and assembled *de novo* with NOVOPlasty v3.6 (Dierckxsens et al. 2017). Coverage analysis revealed an average depth of 2772× (Figure S1), following method from the previous study (Ni et al. 2023). Annotation of mitochondrial genome was performed using MFannot (Lang et al. 2023) and GeSeq (Tillich et al. 2017), with tRNA genes predicted by tRNAscan-SE v2.0 (Lowe and Chan 2016) and manually curated in Geneious Prime v.2025.2.2 (Dotmatics, Boston, MA). A circular genome map was visualized using OGDRAW v 1.3.1 (Greiner et al. 2019). For phylogenetic analysis, 14 conserved protein-coding genes (PCGs) (*atp6*, *atp8*, *atp9*, *cob*, *cox1–3*, *nad1–6*, and *nad4L*) were aligned using MUSCLE v.5.3 (Edgar 2022) and poorly aligned positions were removed with Gblock v. 0.91b (Castresana 2000). Then, each alignment was tested for selecting the substitution model using ModelTest-NG v0.1.7 (Darriba et al. 2020) and all alignments were concatenated to single alignment. For all markers, MTZOA was chosen as best-fit model, except for *cox1* (LG), *nad5* (DCMUT), and *nad6* (JTT). *Clonostachys farinosa* (Henn.) Rossman 2014 (OQ205181) (Oh 2024) and *Emericellopsis fuci* (Summerb., Zuccaro & W. Gams) L.W. Hou, L. Cai & Crous 2023 (KR864757) (Konovalova and Logacheva 2016) belonged to Bionectriaceae in Hypocreales were used as outgroups. Maximum-likelihood phylogenetic analysis was conducted using RAxML v. 8.2.12 (Stamatakis 2014) with 1000 bootstrap replicates in raxmlGUI v2.0 (Edler et al. 2021).

## Results

**Figure 2.**
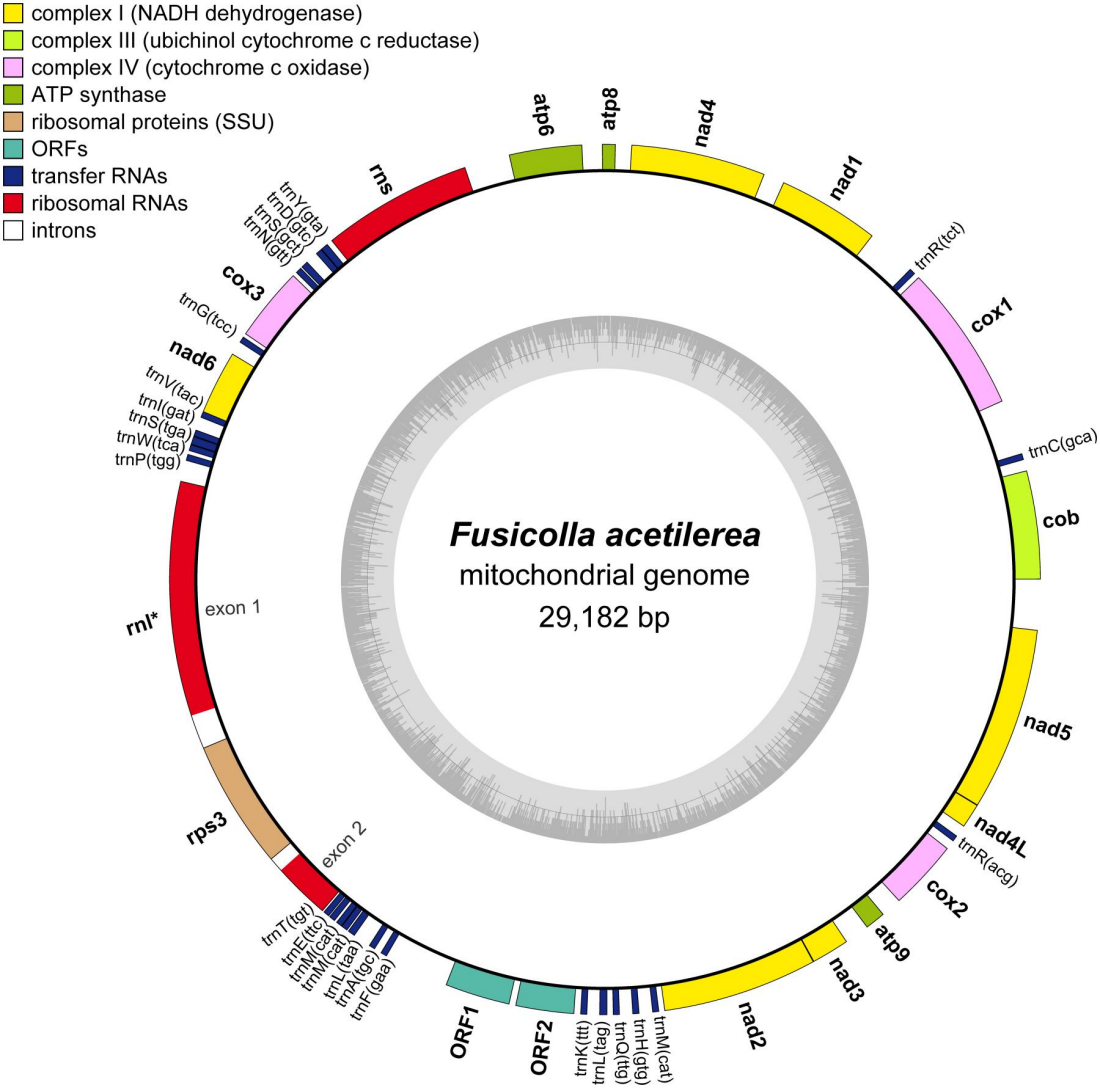
Mitogenome feature maps of *Fusicolla acetilerea* strain NIBRFGC000505922. Genes are depicted on the outer circle and color-coded according to functional categories. GC content is shown in the inner circle. Genes containing introns are indicated with an asterisk (*).

**Figure 3.**
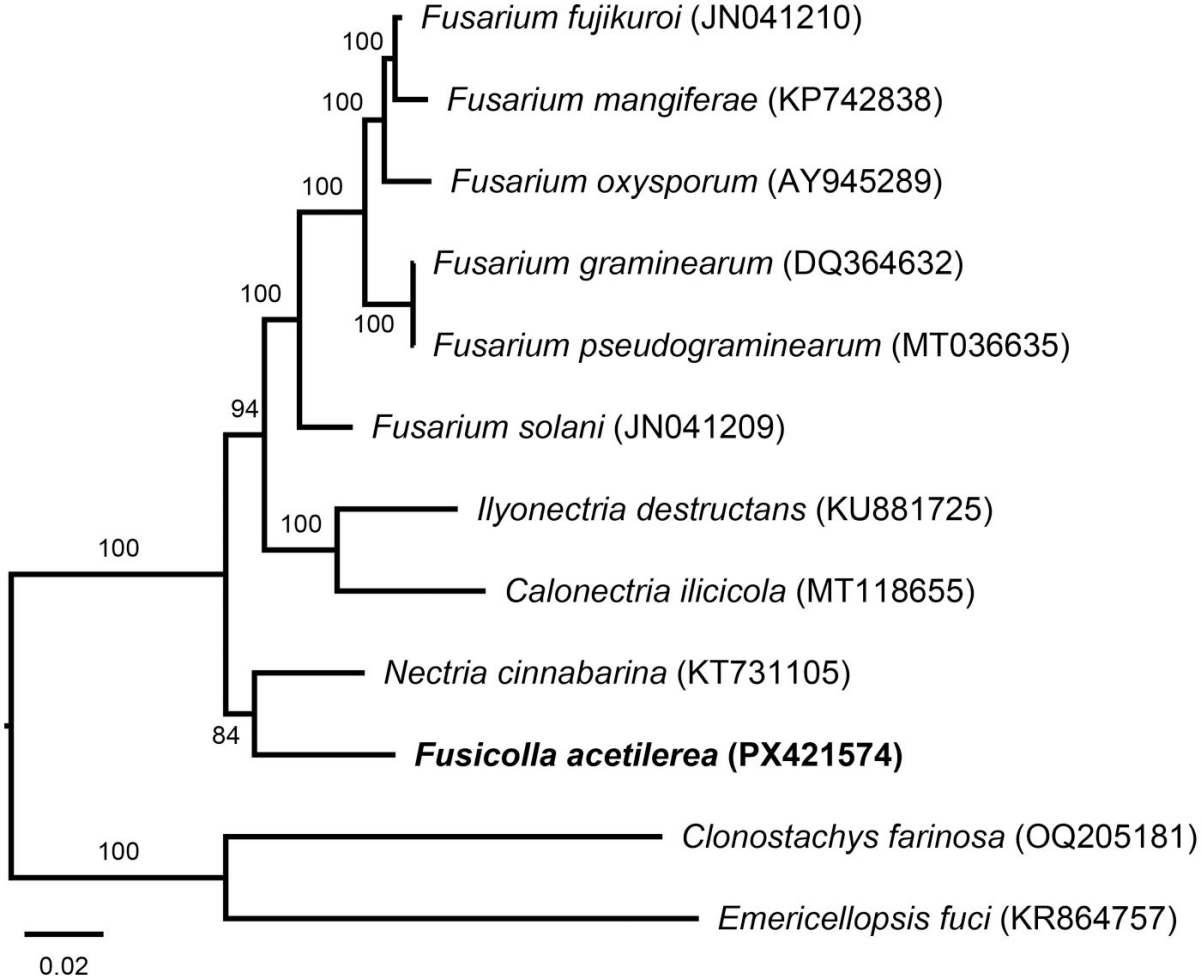
Maximum-likelihood (ML) phylogenetic tree of nine Nectriaceae species inferred from concatenated amino acid sequences of 14 conserved mitochondrial genes (*atp6*, *atp8*, *atp9*, *cob*, *cox1*–*3*, *nad1*–*6*, and *nad4L*). *Calonectria ilicicola* (MT118655.1; Gai et al. 2020), *Clonostachys farinosa* (OQ205181.2; Oh 2024), *Emericellopsis fuci* (KR864757.1; Konovalova and Logacheva 2016), *Fusarium fujikuroi* (JN041210.1; Al-Reedy et al. 2012), *Fusarium graminearum* (DQ364632.1; Al-Reedy et al. 2012), *Fusarium mangiferae* (KP742838.1; unpublished), *Fusarium oxysporum* (AY945289.1; Pantou et al. 2008), *Fusarium pseudograminearum* (MT036635.1; Kulik et al. 2020), *Fusarium solani* (JN041209.1; Al-Reedy et al. 2012), *Ilyonectria destructans* (KU881725.1; Okorski and Majchrzak 2007), and *Nectria cinnabarina* (KT731105.1; Wang et al. 2016) were included as a reference. Bootstrap support values (1000 replicates) are indicated on the branch. The scale bar shows the substitution number per site.

The complete mitochondrial genome of *F. acetilerea* was assembled into a circular molecule of 29,182 bp in length, with an overall GC content of 28.7% (Figure 2). The mitogenome encodes a total of 14 standard PCGs involved in oxidative phosphorylation, including three subunits of cytochrome c oxidase (*cox1*, *cox2*, and *cox3*), apocytochrome b (*cob*), seven subunits of NADH dehydrogenase (*nad1*, *nad2*, *nad3*, *nad4*, *nad4L*, *nad5*, and *nad6*), and three ATP synthase subunits (*atp6*, *atp8*, and *atp9*). Additionally, the genome contains two ribosomal RNA genes (rnl and rns) and 25 transfer RNA genes (tRNAs) corresponding to all 20 standard amino acids, with some tRNAs exhibiting redundancy (Arg, Leu, Met, and Ser). Intron was found in rnl region as a group IA, harboring *rps3* gene. In addition, two free-standing open reading frames (ORF1 and ORF2) encoding hypothetical proteins were annotated in the mitochondrial genome. Phylogenetic analysis based on concatenated amino acid sequences of the 14 PCGs placed *F. acetilerea* within the family Nectriaceae, forming a sister relationship with *Nectria cinnabarina* (Tode) Fr. 1849, with strong bootstrap support (84%) (Figure 3). The mitogenome sequence has been deposited in GenBank under accession number PX421574.

## Discussion and conclusions

The mitogenome of *F. acetilerea* obtained from this study is the first complete mitochondrial genome characterized for the genus *Fusicolla*, marking a significant contribution in the genomic study of Nectriaceae fungi. The conserved gene content, comprising 14 PCGs, two rRNA genes, and 25 tRNA genes, align closely with those reported for related Nectriaceae species (Al-Reedy et al. 2012; Gai et al. 2020). In addition, the presence of a group IA intron in the rnl region encoding the *rps3* gene is similar to patterns observed in other Nectriaceae mitogenomes, consistent with ribosomal protein synthesis regulation in fungal mitochondria (Kouvelis and Hausner 2022). A distinctive feature of the *F. acetilerea* is its compact size of mitogenome (29,182 bp), making it the smallest known mitogenome within the Nectriaceae family (30,629–110,525 bp) (Kulik et al. 2020). The compact mitogenome *of F. acetilerea* may reflect reduced intergenic regions or fewer intronic sequences, though the specific evolutionary factors contributing to this compactness remain unclear. Phylogenetic analysis of mitogenome positioned *F. acetilerea* as a sister to *N. cinnabarina* within Nectriaceae, aligning with recent taxonomic revisions in the family (Lombard et al. 2015). This relationship highlights the utility of mitogenome as robust phylogenetic markers for resolving evolutionary relationships within Nectriaceae.

The significance of this study is amplified by the limited availability of mitogenomic data within Nectriaceae. Although multiple *Fusarium* species have sequenced mitogenomes, most other genera remain unrepresented, except for *Calonectria* and *Nectria* with a single species of mitogenome (Wang et al. 2016; Gai et al. 2020). This scarcity hinders comparative analyses and evolutionary studies. As the first *Fusicolla* mitogenome, this sequence provides a key reference for studying mitochondrial evolution and intron dynamics within Nectriaceae.

## Supplementary Material

Supplemental Material

## Data Availability

The sequence generated from this study has been deposited to GenBank under accession number PX421574. The associated BioProject, BioSample, and SRA numbers are PRJNA1301341, SAMN50439991, and SRR35602807, respectively.

## References

[CIT0001] Al-Reedy RM, Malireddy R, Dillman CB, Kennell JC. 2012. Comparative analysis of *Fusarium* mitochondrial genomes reveals a highly variable region that encodes an exceptionally large open reading frame. Fungal Genet Biol. 49(1):2–14. 10.1016/j.fgb.2011.11.008 22178648

[CIT0002] Asano Y, Ando S, Tani Y, Yamada H, Ueno T. 1981. Fungal degradation of triacrylonitrile. Agric Biol Chem. 45(1):57–62. 10.1080/00021369.1981.10864495

[CIT0003] Biedermann PH, Klepzig KD, Taborsky M, Six DL. 2013. Abundance and dynamics of filamentous fungi in the complex ambrosia gardens of the primitively eusocial beetle *Xyleborinus saxesenii* Ratzeburg (Coleoptera: Curculionidae, Scolytinae). FEMS Microbiol Ecol. 83(3):711–723. 10.1111/1574-6941.12026 23057948

[CIT0004] Castresana J. 2000. Selection of conserved blocks from multiple alignments for their use in phylogenetic analysis. Mol Biol Evol. 17(4):540–552. 10.1093/oxfordjournals.molbev.a026334 10742046

[CIT0005] Chen S, Zhou Y, Chen Y, Gu J. 2018. fastp: an ultra-fast all-in-one FASTQ preprocessor. Bioinformatics. 34(17):i884–i890. 10.1093/bioinformatics/bty560 30423086 PMC6129281

[CIT0006] Darriba D et al. 2020. ModelTest-NG: a new and scalable tool for the selection of DNA and protein evolutionary models. Mol Biol Evol. 37(1):291–294. 10.1093/molbev/msz189 31432070 PMC6984357

[CIT0007] Dierckxsens N, Mardulyn P, Smits G. 2017. NOVOPlasty: de novo assembly of organelle genomes from whole genome data. Nucleic Acids Res. 45(4):e18. 10.1093/nar/gkw955 28204566 PMC5389512

[CIT0008] Ding S, Hu H, Gu JD. 2015. Fungi colonizing wood sticks of Chinese fir incubated in subtropical urban soil growing with *Ficus microcarpa* trees. Int J Environ Sci Technol. 12(12):3781–3790. 10.1007/s13762-015-0802-5

[CIT0009] Edgar RC. 2022. Muscle5: high-accuracy alignment ensembles enable unbiased assessments of sequence homology and phylogeny. Nat Commun. 13(1):6968. 10.1038/s41467-022-34630-w 36379955 PMC9664440

[CIT0010] Edler D, Klein J, Antonelli A, Silvestro D. 2021. raxmlGUI 2.0: a graphical interface and toolkit for phylogenetic analyses using RAxML. Methods Ecol Evol. 12(2):373–377. 10.1111/2041-210x.13512

[CIT0011] Fujimitsu H et al. 2016. Purification and characterization of 4-N-trimethylamino-1-butanol dehydrogenase *from Fusarium merismoides* var. *acetilereum*. Biosci Biotechnol Biochem. 80(9):1753–1758. 10.1080/09168451.2016.1177443 27121905

[CIT0012] Gai Y, Pan R, Peng X. 2020. A phylogenomic tree of fungi: evolutionary relationships among *Calonectria ilicicola* and 586 fungal mitochondrial genomes. Mitochondrial DNA Part B. 5(2):1709–1711. 10.1080/23802359.2020.1749163

[CIT0013] Gräfenhan T, Schroers HJ, Nirenberg HI, Seifert KA. 2011. An overview of the taxonomy, phylogeny, and typification of nectriaceous fungi in *Cosmospora*, *Acremonium*, *Fusarium*, *Stilbella*, and *Volutella*. Stud Mycol. 68(1):79–113. 10.3114/sim.2011.68.04 21523190 PMC3065986

[CIT0014] Greiner S, Lehwark P, Bock R. 2019. OrganellarGenomeDRAW (OGDRAW) version 1.3.1: expanded toolkit for the graphical visualization of organellar genomes. Nucleic Acids Res. 47(W1):W59–W64. 10.1093/nar/gkz238 30949694 PMC6602502

[CIT0015] Jeon YJ, Goh J, Mun HY. 2020. Diversity of fungi in brackish water in Korea. Korean J Mycol. 48(4):457–473.

[CIT0016] Konovalova O, Logacheva M. 2016. Mitochondrial genome of two marine fungal species. Mitochondrial DNA A DNA Mapp Seq Anal. 27(6):4280–4281. 10.3109/19401736.2015.1082094 26703096

[CIT0017] Kouvelis VN, Hausner G. 2022. Mitochondrial genomes and mitochondrion related gene insights to fungal evolution. Front Microbiol. 13:897981. 10.3389/fmicb.2022.897981 35479620 PMC9036184

[CIT0018] Kulik T et al. 2020. Diversity of mobile genetic elements in the mitogenomes of closely related *Fusarium culmorum* and *F. graminearum* sensu stricto strains and its implication for diagnostic purposes. Front Microbiol. 11:1002. 10.3389/fmicb.2020.01002 32528440 PMC7263005

[CIT0019] Lang BF et al. 2023. Mitochondrial genome annotation with MFannot: a critical analysis of gene identification and gene model prediction. Front Plant Sci. 14:1222186. 10.3389/fpls.2023.1222186 37469769 PMC10352661

[CIT0020] Lechat C, Rossman AY. 2017. A new species of *Fusicolla* (Hypocreales), *F. ossicola*, from Belgium. Ascomycete.org. 9(6):225–228.

[CIT0021] Lee SY, Ten LN, Das K, You YH, Jung HY. 2021. Biodegradative activities of fungal strains isolated from terrestrial environments in Korea. Mycobiology. 49(3):285–293. 10.1080/12298093.2021.1903131 36999090 PMC10049743

[CIT0022] Liu C, Zhuang WY, Yu ZH, Zeng ZQ. 2022. Two new species of *Fusicolla* (Hypocreales) from China. Phytotaxa. 536(2):165–174. 10.11646/phytotaxa.536.2.5

[CIT0023] Lombard L, Van der Merwe NA, Groenewald JZ, Crous PW. 2015. Generic concepts in Nectriaceae. Stud Mycol. 80(1):189–245. 10.1016/j.simyco.2014.12.002 26955195 PMC4779799

[CIT0024] Lowe TM, Chan PP. 2016. tRNAscan-SE On-line: integrating search and context for analysis of transfer RNA genes. Nucleic Acids Res. 44(W1):W54–W57. 10.1093/nar/gkw413 27174935 PMC4987944

[CIT0025] Ni Y, Li J, Zhang C, Liu C. 2023. Generating sequencing depth and coverage map for organelle genomes. protocols.io. 10.17504/protocols.io.4r3l27jkxg1y/v1

[CIT0026] Oh SY. 2024. The complete mitochondrial genome of *Clonostachys farinosa* (Bionectriaceae, Hypocreales). Mitochondrial DNA B Resour. 9(5):583–587. 10.1080/23802359.2024.2347510 38716395 PMC11075654

[CIT0027] Okorski A, Majchrzak B. 2007. Fungi isolated from soil before the seeding and after harvest of pea [*Pisum sativum* L.] after application of bio-control product EM 1. Acta Agrobot. 60(1):113–121. 10.5586/aa.2007.014

[CIT0028] Pantou MP, Kouvelis VN, Typas MA. 2008. The complete mitochondrial genome of *Fusarium oxysporum*: insights into fungal mitochondrial evolution. Gene. 419(1–2):7–15. 10.1016/j.gene.2008.04.009 18538510

[CIT0029] Stamatakis A. 2014. RAxML version 8: a tool for phylogenetic analysis and post-analysis of large phylogenies. Bioinformatics. 30(9):1312–1313. 10.1093/bioinformatics/btu033 24451623 PMC3998144

[CIT0030] Tillich M et al. 2017. GeSeq – versatile and accurate annotation of organelle genomes. Nucleic Acids Res. 45(W1):W6–W11. 10.1093/nar/gkx391 28486635 PMC5570176

[CIT0031] Tubaki K, Booth C, Harada T. 1976. A new variety of *Fusarium merismoides*. Trans Br Mycol Soc. 66(2):355–356. 10.1016/S0007-1536(76)80072-1

[CIT0032] Wang M et al. 2020. *Streptomyces lydicus* M01 regulates soil microbial community and alleviates foliar disease caused by *Alternaria alternata* on cucumbers. Front Microbiol. 11:942. 10.3389/fmicb.2020.00942 32499771 PMC7243425

[CIT0033] Wang XC, Zeng ZQ, Zhuang WY. 2016. The complete mitochondrial genome of the important phytopathogen *Nectria cinnabarina* (Hypocreales, Ascomycota). Mitochondrial DNA A DNA Mapp Seq Anal. 27(6):4670–4671. 10.3109/19401736.2015.1106495 27159696

[CIT0034] Zeng ZQ, Zhuang WY. 2023. Three new species of *Fusicolla* (Hypocreales) from China. J Fungi. 9(5):572. 10.3390/jof9050572 PMC10219165 37233283

[CIT0035] Zhong M et al. 2021. *Candida albicans* disorder is associated with gastric carcinogenesis. Theranostics. 11(10):4945–4956. 10.7150/thno.55209 33754037 PMC7978306

[CIT0036] Zhu ZY, Huang YG. 2021. Structure and diversity analysis of mold community in main Maotai-flavor Baijiu brewing areas of Maotai town using high-throughput sequencing. Shipin Kexue/Food Sci. 42(8):150–156.

