# Supplementary figures and images for: The complete mitochondrial genome of Fusicolla acetilerea (Nectriaceae, Hypocreales)
Source: Mitochondrial DNA B Resour. 2026 Feb 16;11(3):419–23. doi: 10.1080/23802359.2026.2630474 (PMC12912206; doi:10.1080/23802359.2026.2630474)

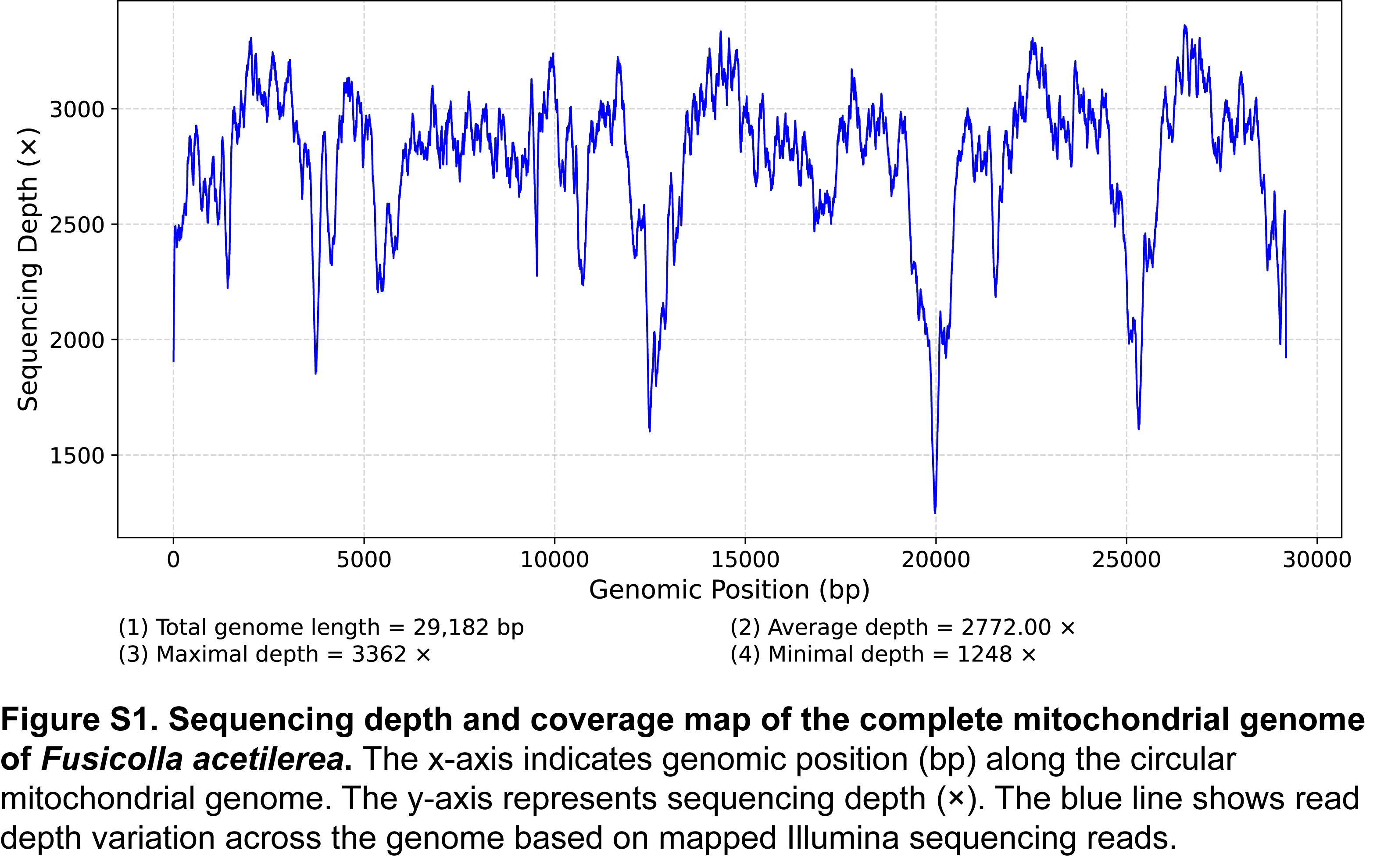

Supplement: Supplemental Material [file TMDN_A_2630474_SM8797.tif]
